# PLACES: Local Data for Better Health

**DOI:** 10.5888/pcd19.210459

**Published:** 2022-06-16

**Authors:** Kurt J. Greenlund, Hua Lu, Yan Wang, Kevin A. Matthews, Jennifer M. LeClercq, Benjamin Lee, Susan A. Carlson

**Affiliations:** 1 Division of Population Health, National Center for Chronic Disease Prevention and Health Promotion, Centers for Disease Control and Prevention, Atlanta, Georgia; 2 Oak Ridge Institute for Science and Education, Research Participation Program, Division of Population Health, Centers for Disease Control and Prevention, Atlanta, Georgia

## Abstract

Local-level data on the health of populations are important to inform and drive effective and efficient actions to improve health, but such data are often expensive to collect and thus rare. Population Level Analysis and Community EStimates (PLACES) (www.cdc.gov/places/), a collaboration between the Centers for Disease Control and Prevention (CDC), the Robert Wood Johnson Foundation, and the CDC Foundation, provides model-based estimates for 29 measures among all counties and most incorporated and census-designated places, census tracts, and ZIP Code tabulation areas across the US. PLACES allows local health departments and others to better understand the burden and geographic distribution of chronic disease–related outcomes in their areas regardless of population size and urban–rural status and assists them in planning public health interventions. Online resources allow users to visually explore health estimates geographically, compare estimates, and download data for further use and exploration. By understanding the PLACES overall approach and using the easy-to-use PLACES applications, practitioners, policy makers, and others can enhance their efforts to improve public health, including informing prevention activities, programs, and policies; identifying priority health risk behaviors for action; prioritizing investments to areas with the biggest gaps or inequities; and establishing key health objectives to achieve community health and health equity.

**Summary**

**What is already known on this topic?**

Local-level population health data are important for effective planning and resource allocation, yet such data are rarely available for most geographic areas below the state level.

**What is added by this report?**

The PLACES platform provides a set of 29 chronic disease–related measures for all US counties and most incorporated and census-designated places, census tracts, and ZIP Code tabulation areas using the same methodology.

**What are the implications for public health practice?**

By understanding the overall approach and using available resources and applications for PLACES, practitioners, policy makers, and other users can further support their efforts to improve health.

## Introduction

Chronic diseases comprise 7 of the 10 leading causes of death in the US (1), and approximately half of US adults have at least 1 chronic condition (2). The extent of chronic disease in the population and recent public health emergencies, such as the COVID-19 pandemic and natural disasters (3–5), highlight the need for prevention and management efforts for people with chronic conditions and their risk factors.

Standardized and reliable health data for local areas across the US are critical to advancing health for all Americans, because implementing interventions and policies to promote health are largely driven at the local level. Effective planning and decision making rely on credible health information. Having detailed local information can identify patterns in the prevalence of disease as well as geographic disparities. Although planners in some areas of the US have access to geographically detailed data about chronic conditions and risk-related behaviors for their jurisdictions, most do not. One reason for the lack of local health data is the cost of directly collecting information to assess the health of populations, including data to assess and act on factors that differentially affect the health of population groups. In the absence of resources to directly collect information on the health of local populations, various statistical methods can provide alternative estimates of health measures and help conserve scarce resources for prevention activities. Beginning in 2015, the Centers for Disease Control and Prevention (CDC), with support from the Robert Wood Johnson Foundation and the CDC Foundation, implemented a small area estimation (SAE) approach that provided chronic disease–related health measures for large cities and their census tracts in the 500 Cities project (www.cdc.gov/places/about/500-cities-2016-2019/index.html). This effort was expanded in 2020 to cover all counties and all incorporated and census-designated places, census tracts, and ZIP Code Tabulation Areas (ZCTAs) with a population of 50 or more and renamed PLACES (Population Level Analysis and Community EStimates) (www.cdc.gov/places/). PLACES provides first-of-their-kind US population-level analyses and community estimates, including data for small cities and rural areas — data that were previously unavailable.

## Approach to Small Area Estimation

SAE generally refers to a family of statistical procedures that estimates measures for a small geographic area by using the observed information collected for a larger area. The data for the large area can be survey data, vital statistics, or disease registry data. A variety of model-based SAE methods have been developed (6–8), and approaches vary based on the purpose of the analyses.

In response to frequent requests for population health data at multiple geographic or administrative levels (eg, county, health services area, congressional districts, cities, metropolitan areas) in a uniform and efficient manner, the CDC Division of Population Health, beginning in the 2000s, adapted an SAE approach called multilevel regression and post stratification (MRP), which was initially developed to estimate state-level voter preference by using national polls (9). The program extended this approach to the census-block level, which could then be aggregated to any larger geographic unit on the basis of census blocks by using state-level health data from the Behavioral Risk Factor Surveillance System (BRFSS) (10). After several years of refining and validating the approach (11–14), the program collaborated with the Robert Wood Johnson Foundation and the CDC Foundation to produce estimates for adult chronic disease–related measures, initially for the 500 largest US cities and their census tracts, covering 33.4% of the US adult population. In 2020 the project was expanded and renamed PLACES to provide prevalence estimates at 4 geographic levels across the US: county, place (incorporated and census-designated), census tract, and ZCTA.

Estimates are currently available for 29 health measures (Table): 13 health outcomes, 4 health risk behaviors, 9 prevention practices, and 3 health status measures, capturing risk factors, behaviors, and conditions that have a substantial impact on population health. Measures of diagnosed depression and general health status were added with the 2021 data release. The measures were chosen because they contribute substantially to chronic disease morbidity and mortality rates and are amenable to intervention. Other factors, such as how often a particular BRFSS question was asked, were also considered when choosing these measures. The measure definitions reflect the work of the Chronic Disease Indicators Project, a collaboration of CDC, the Council of State and Territorial Epidemiologists, and the National Association of Chronic Disease Directors, to develop a uniform set of data measurement definitions (15,16).

**Table:** Chronic Disease–Related Measures Included in PLACES^a^, 2021

Health outcomes	Health risk behaviors	Preventive services	Health status
Arthritis Current asthma High blood pressure Cancer (excluding skin cancer) High cholesterol (among those screened in the past 5 years) Chronic kidney disease Chronic obstructive pulmonary disease Coronary heart disease Diagnosed diabetes All teeth lost, adults aged ≥65 years Stroke Obesity Diagnosed depression	Binge drinking Current smoking No leisure-time physical activity Sleeping less than 7 hours	Current lack of health insurance (aged 18–64 years) Visits to doctor for routine checkup within the past year Visits to dentist or dental clinic Taking medicine for high blood pressure control (among adults with high blood pressure) Cholesterol screening Mammography screening (women aged 50–74 years) Cervical cancer screening (women aged 21–65 years) Fecal occult blood test, sigmoidoscopy, or colonoscopy (aged 50–75 years) Adults aged ≥65 years who are up to date on a core set of clinical preventive services (separate measures for women and men)	Mental health not good for ≥14 days past month Physical health not good for ≥14 days past month Fair/poor general health status

Abbreviation: PLACES, Population Level Analysis and Community EStimates.

a www.cdc.gov/places/.

Here, we provide a background to understanding the data, discuss available tools for exploring the data, and provide some examples of how the data can be used for action.

## Current Estimation Approach

**Modeling approach**. A multilevel logistic regression model is constructed for each outcome, which includes individual-level age (13 categories), sex (2 categories), race or ethnicity (8 categories), and education (4 categories) from BRFSS; county-level percentage of adults below 150% of the federal poverty level from the 5-year American Community Survey (17); and state- and county-level random effects. To estimate the predicted probability for the risk of each measure, model parameters are applied to the same age–race or ethnicity–sex categories (n = 208) for each small area by using the multilevel logistic model formula. The predicted probability for each measure is multiplied by the corresponding population for each area to produce its estimated (or expected) prevalence. Concurrent population estimates were used to create the county estimates (18), and the 2010 decennial population counts were used to create estimates for incorporated and census-designated places, census tracts, and ZCTAs (19). Because these population estimates or counts cross-tabulated by education level are not available, a bootstrapping method assigns education level to each population category for each small area. To obtain the distribution of the estimate, a Monte Carlo simulation is used to draw 1,000 samples of the model parameters from their estimated conditional distributions to generate a sample of 1,000 SAEs for each small area; the final estimate for each measure is reported as the mean and 95% CI (2.5th and 97.5th percentile) of these 1,000 samples for all 3,142 US counties and for 28,484 incorporated and census-designated places, 72,337 census tracts, and 32,409 ZCTAs.

**Strengths**. PLACES provides a uniformly developed set of local-level chronic disease estimates based on standardized definitions that can be used for health planning. Our approach combines both individual and area-specific information relevant to small area estimation of population health outcomes. Modeling nationally and predicting locally can help fill the need for local data, especially where direct local data are unavailable. PLACES data can be used by state and local health officials, policy makers, nonprofit organizations, and others to advance health by:

- Informing the development and implementation of effective and tailored prevention activities, programs, and policies
- Identifying emerging health problems and priority health risk behaviors for action
- Understanding the prevalence and geographic distribution of health-related issues and prioritizing investment in areas with the biggest gaps or inequities
- Establishing key health objectives that the community can focus on to improve health

**Limitations and cautions**. First, the modeling process does not control for known potential biases in self-reported surveys such as BRFSS, including recall or social desirability biases. BRFSS is the largest telephone-based (landline and cellular telephone) survey of health behaviors and factors in the world (10). BRFSS has been shown to produce estimates generally comparable to national surveys (20-22) and is a critical — and, in many cases, a primary — data source for state-level estimates of many chronic disease–related measures. Second, detailed population count data with the relevant stratifications needed for implementing our approach are available only for the decennial census year. Therefore, incorporated and census-designated place, census tract, and ZCTA-level estimates are currently based on 2010 census population counts. For county-level estimates, vintage population estimates released by the US Census Bureau (18) that match the year of the BRFSS data are used. Third, the model does not consider any local policy or intervention effects that may affect area-specific estimates.

The lack of comparable local health data prohibits extensive validation of the PLACES MRP approach, particularly for areas smaller than counties. However, validation studies have observed that the PLACES model-based estimates show strong correlations with direct survey estimates from both BRFSS data and local survey data at the state, county, and city levels (11–14). These studies suggest that correlations are acceptable but vary by geographic level and indicator when assessed against comparable data from sources other than BRFSS, which should be expected because of different methods and measurement errors in other data sets (13,14). Where reliable, directly measured data are available, such data should be considered.

Because the model relies on decennial census data for small geographies, we caution against using the estimates for assessing changes or evaluating intervention effects over time. Also, because the statistical approach incorporates age, race or ethnicity, sex, education, and poverty to generate the estimates, others using these estimates should use caution when including PLACES estimates in other models, because some statistical collinearity is possible. Although PLACES shows geographic disparities in health, currently it does not display estimates by race or ethnicity or sex within or across geographies. However, assessing population health needs by examining the demographic composition within and across areas is appropriate. For example, PLACES estimates can be used to broadly assess health disparities across geographic areas and then examine the demographic composition of those areas to describe the populations to whom the estimates may apply, for example, whether an area is poorer, older, or has a large minority population. In addition to mapping functions such as highlighting rural and urban areas, other visualizations such as charts and graphs can complement maps. Although a focus may be on poor health, comparing healthy populations to maintain or promote health is also appropriate.

## PLACES Applications and Online Resources

The PLACES website provides resources, including measure definitions, methodology, references to publications, frequently asked questions, communication tools such as fact sheets and multimedia graphics, and help pages for using the website and applications. To meet the diverse needs of users, data can be obtained, visualized, or downloaded by using various tools and applications. CDC data hosted by Socrata on CDC.gov has a feature that allows users to easily embed PLACES data on their own website.

**Figure 1:**
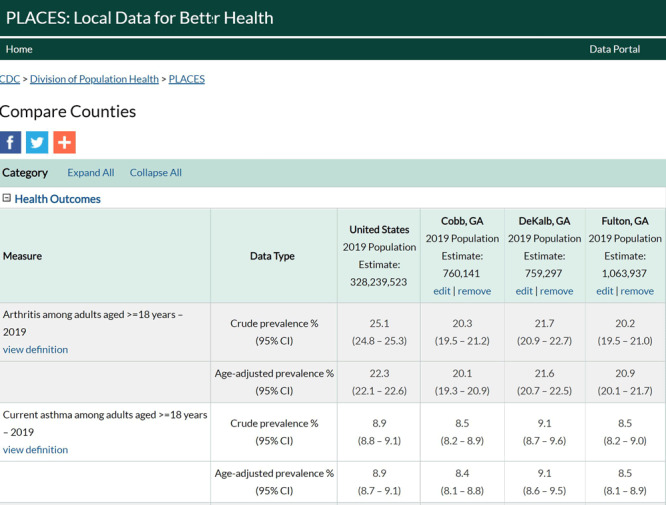
Screen shot of a PLACES Compare Counties Report comparing data for 3 Georgia counties and the US overall. Users can choose and compare data between the US and up to 3 counties.

**Compare Counties Report**. This report allows users to compare data between the US and up to 3 counties. By default, all categories (health outcomes, prevention, health risk behaviors, health status) display fully expanded to show data for all measures at the national level (default location is the US). Crude and age-adjusted prevalence and their corresponding CIs appear for each measure. For example, Figure 1 shows a Compare Counties Report of 3 neighboring counties in the Atlanta area. In addition to the crude and age-adjusted estimates for each measure, the population size for each county is listed. A more in-depth help section for using the Compare Counties Report is provided (www.cdc.gov/places/help/explore-compare-counties-report/index.html).

**Figure 2:**
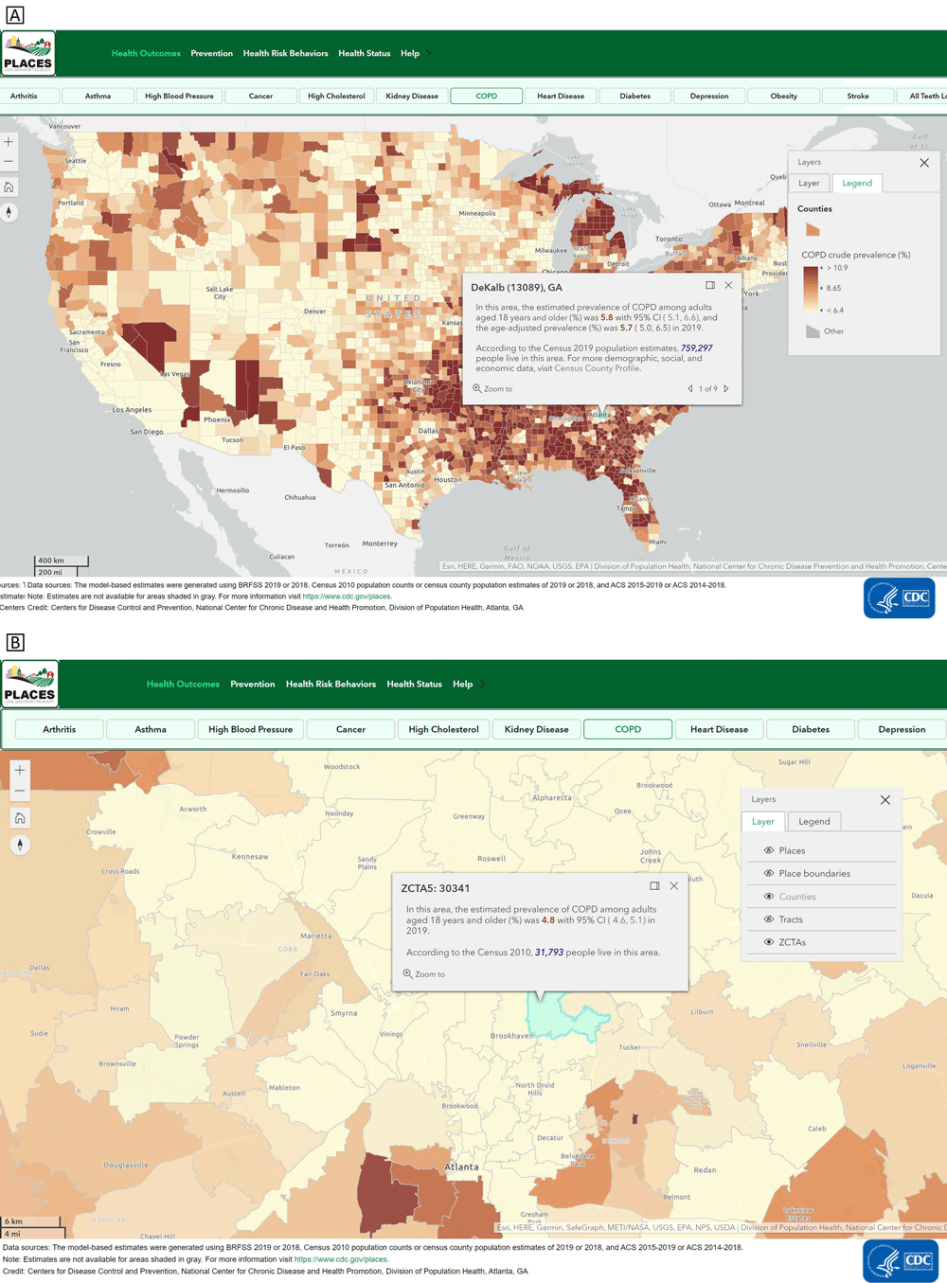
PLACES interactive map application (www.cdc.gov/PLACES). Users can examine and visualize health data estimates across different geographic levels by using the PLACES interactive mapping application. By clicking a specific location, the selected measure (eg, estimated prevalence and crude prevalence) will appear for the selected chronic disease at that location. By zooming in and clicking on a particular geographic area, users can view the estimate for smaller geographic units. In Figure 2A, the county-level prevalence of COPD in Dekalb County, Georgia, is shown. Figure 2B displays COPD prevalence estimates at the ZIP Code tabulation area (ZTCA), which can be discerned by looking at the layer tool. Abbreviations: COPD, chronic obstructive pulmonary disease; PLACES, Population Level Analysis and Community EStimates.

**Interactive mapping**. Users can examine and visualize health estimates across different geographic levels by using the interactive mapping application. For example, the map in Figure 2A displays estimates of chronic obstructive pulmonary disease (COPD) at the county level. By clicking a specific location, the estimate for that location will appear. The map shows that the county-level prevalence of COPD is 5.8% in DeKalb County, Georgia. The mean county-level prevalence of COPD is 8.65%, as shown in the figure legend. By zooming in and clicking on a particular geographic area, users can view the estimate for smaller geographic units. Figure 2B displays COPD prevalence estimates at the ZCTA level, which can be discerned by looking at the layer tool. Estimated COPD prevalence for ZCTA 30341 is 4.8%. A more in-depth help section for using the interactive map is provided (www.cdc.gov/places/help/explore-interactive-map/index.html).

Advanced users can access ArcGIS Online service (Esri) and the individual maps for each measure available in CDC’s gallery of the ArcGIS Living Atlas of the World (23). Most measures have a single map, except for the core preventive services use measure, which has separate maps for older men and women. These web maps allow users to zoom to specific areas, filter data, use analysis tools, and overlay with other data available on the ArcGIS Living Atlas of the World (for example, American Community Survey socioeconomic data) or their own data.

**Downloading data**. Estimates can be downloaded, enabling users to view, search, filter, and further use and analyze the data. Data can be downloaded for all 4 geographic levels by using the data portal (https://chronicdata.cdc.gov/browse?category=500+Cities+%26+Places). Data are available in 2 formats: 1 measure per row (open data) or all measures per row (GIS-friendly). In addition to downloading data, estimates can be viewed as tables and searched or filtered through the data portal. A more in-depth help section for using the data portal is provided (www.cdc.gov/places/help/explore-data-portal/index.html).

## PLACES Methods and Data in Action

In the first year (December 7, 2020–December 3, 2021), PLACES received more than 200,000 page views from approximately 64,000 unique visitors. To illustrate the usefulness of PLACES, we provide some examples of how the PLACES approach and data are being used to support public health action.

The PLACES MRP approach has been applied to several public health issues (24-32). The COVID-19 pandemic highlighted the susceptibility of people with chronic conditions to severe illness and outcomes from other diseases like COVID-19. People with certain chronic conditions were observed to have higher risks of severe outcomes from COVID-19 (33). By using the PLACES MRP methods, the number and percentage of US adults with specific chronic conditions (heart disease, COPD, diabetes, chronic kidney disease, obesity) and with at least 1 of those conditions were estimated. The county level prevalence of at least 1 of these 5 conditions ranged from almost 1 in 4 adults to as many as 2 in 3 adults (3). Estimates were provided on the CDC COVID Data Tracker (34) as a visual tool to help local mitigation efforts. Findings highlighted the need for prevention and control of chronic conditions and the importance of chronic conditions and risk factors in prevention and mitigation efforts for infectious disease outbreaks such as COVID-19. Providing the estimates in an interactive format allows local decision makers to identify areas at high risk for severe COVID-19 illness in their jurisdictions and guide resource allocation and implementation of community mitigation strategies.

PLACES data have been combined with other data to identify and plan for at-risk populations, including before, during, and after natural disasters or other emergencies. The prevalence of most chronic conditions does not change quickly over time, so estimates such as those provided in PLACES could be used in a near real-time manner when combined with other data that may change quickly over time. For example, it was demonstrated that the estimated number and percentage of people with COPD residing in census tracts within 50, 100, and 200 miles of the path of Hurricane Florence that struck the Carolinas in 2018 could be quickly calculated and re-calculated as the predicted hurricane path changed; such information can help to anticipate, respond to, and ameliorate health threats in preparedness and response efforts (5).

PLACES estimates are incorporated into multiple CDC websites (35-37) and have been incorporated into other external applications, including County Health Rankings and Roadmaps (38) and the City Health Dashboard (39). These platforms bring together PLACES data with other data sources while also providing guidance on actions to address health. For example, the County Health Rankings and Roadmaps website provides tools and guidance on taking action, information on evidence-informed policies and programs, and tips on identifying and working with partners. PLACES data also have been incorporated into specific state- and local-level websites. For example, PLACES data are included as part of the Hawai’i Health Matters website, a one-stop source of information about community health on the islands (40).

Finally, PLACES has been used to prioritize investment to areas with the largest health gaps or inequities. CDC’s Division of Nutrition, Physical Activity, and Obesity used 2015 county-level estimates of obesity derived from the PLACES approach to determine eligibility for the High Obesity Program (41).

## PLACES of the Future

PLACES provides previously unavailable local-level health estimates through a standardized and validated SAE approach with the goal of supporting public health planning and action.

Beyond identifying geographic health disparities, PLACES data can be particularly useful when combined with data related to social determinants of health (SDOH) to understand community health and to promote health equity and equal access to health (https://www.cdc.gov/chronicdisease/programs-impact/sdoh.htm). When examined together, users can identify which health and SDOH issues overlap in a community to inform and shape public health actions. For example, identifying communities with high rates of chronic disease and high social vulnerability can highlight geographic areas especially in need of strategies tailored for the conditions and risks residents experience. Combining and overlaying data can inform planning activities for partners across sectors like education, transportation, and housing that have the shared goal of improving the conditions in people’s environments.

Using health data such as PLACES with SDOH factors can better align population-based public health approaches and tie in the social factors that contribute to both the causes of health problems and the success of solutions for population health. Beyond the built environment, SDOH includes assessing how structural policies and practices differentially affect the health of populations. With an important public health focus on SDOH and health equity (www.cdc.gov/places/social-determinants-of-health-and-places-data/index.html), further data on social and structural factors that underlie health may become available in future PLACES releases. For example, recent BRFSS surveys include modules on SDOH and on Reactions to Race, which can be assessed and made available if validated. However, PLACES is already in a format for use with such data to explore this critical area.

## Conclusion

The multiple available tools and resources for PLACES allow public health practitioners and others to visualize local chronic disease–related estimates and to download data sets directly from the data portal for health planning and action. By understanding the PLACES approach and improving awareness of the easy-to-use PLACES applications, practitioners, policy makers, and other users can employ these data and tools to inform actions to improve health.
